# A new heterogeneous family of telomerically encoded *Cryptosporidium* proteins

**DOI:** 10.1111/j.1752-4571.2012.00277.x

**Published:** 2012-06-14

**Authors:** Maha Bouzid, Paul R Hunter, Vincent McDonald, Kristin Elwin, Rachel M Chalmers, Kevin M Tyler

**Affiliations:** 1 Biomedical Research Centre, Norwich Medical School, University of East AngliaNorwich, UK; 2 Barts and the London School of Medicine and Dentistry, Blizard Institute of Cell and Molecular Science, Centre for Digestive DiseasesLondon, UK; 3 UK Cryptosporidium Reference Unit, Public Health Wales, Microbiology, Singleton HospitalSwansea, UK

**Keywords:** bioinfomatics/phyloinfomatics, biomedicine, genomics/proteomics, host parasite interactions, microbial biology, molecular evolution, parasitology, virulence

## Abstract

Cryptosporidiosis is predominantly caused by two closely related species of protozoan parasites the zoonotic *Cryptosporidium parvum* and anthroponotic *Cryptosporidium hominis* which diverge phenotypically in respect to host range and virulence. Using comparative genomics we identified two genes displaying overt heterogeneity between species. Although initial work suggested both were species specific, *Cops-1* for *C. parvum* and *Chos-1* for *C. hominis*, subsequent study identified an abridged ortholog of *Cops-1* in *C. hominis*. *Cops-1* and *Chos-1* showed limited, but significant, similarity to each other and share common features: (i) telomeric location: *Cops-1* is the last gene on chromosome 2, whilst *Chos-1* is the first gene on chromosome 5, (ii) encode circa 50-kDa secreted proteins with isoelectric points above 10, (iii) are serine rich, and (iv) contain internal nucleotide repeats. Importantly, *Cops-1* sequence contains specific SNPs with good discriminatory power useful epidemiologically. *C. parvum*-infected patient sera recognized a 50-kDa protein in antigen preparations of *C. parvum* but not *C. hominis*, consistent with Cops-1 being antigenic for patients. Interestingly, anti-Cops-1 monoclonal antibody (9E1) stained oocyst content and sporozoite surface of *C. parvum* only. This study provides a new example of protozoan telomeres as rapidly evolving contingency loci encoding putative virulence factors.

## Introduction

Pathogens can be defined as microbes capable of causing damage to the host, and virulence factors defined as mediators which enable that damage to occur (Casadevall and Pirofski 2001). By these definitions, determinants of host specificity are not only encoded by contingency genes adapted through rapid, red-queen evolution for the particular biological niche represented by their host, but are virulence factors which specifically enable colonization, infection and pathogenesis.

Cryptosporidiosis is one of the commonest parasitic causes of diarrhoea and a sometimes fatal disease in the immune-suppressed (Hunter and Nichols 2002; Karanis et al. 2007) with chronic sequelae postacute infection (Hunter et al. 2004). Of some eight species which infect humans (Xiao and Fayer 2008), *Cryptosporidium parvum* and *Cryptosporidium hominis* are the main species of public health importance, causing the majority of human cases as both sporadic and outbreak-related cases. In a survey of over 4000 isolates in the UK, *C. parvum* and *C. hominis* were responsible of 38.5% and 57.3% of the cases, respectively (Chalmers et al. 2010). Although closely related *C. hominis* mainly infects humans, while *C. parvum* exhibits a broader host range including humans, livestock and rodents (Xiao et al. 2004).

The genetic determinants driving the host preference of *Cryptosporidium* species are not yet elucidated, but in other protozoan parasites, such genes are frequently found clustered close to the telomeres, can be hypervariable with regard to sequence and are often part of heterogenous multigene families (Barry et al. 2003). The proteins encoded are frequently surface, shed or secreted glycoproteins and for closely related protozoan parasite isolates these can be the only loci which vary dramatically in terms of allele number and sequence (Franzen et al. 2011).For *Cryptosporidium*, it had been speculated that genome sequencing would assist in uncovering mechanisms of host specificity and virulence; however, this has been hindered by the high sequence similarity (95–97%) between the genome sequences of *C. parvum* and *C. hominis* (Widmer et al. 2002).It has been concluded that the genomes of *C. parvum* and *C. hominis* are highly similar, and the phenotypic differences, which relate mainly to host preference and pathogenicity, are caused by polymorphisms in coding regions and differences in gene regulation (Xu et al. 2004; Pain et al. 2005). This similarity has also largely frustrated reverse diagnostic approaches to the provision of improved tests for robust molecular and serological discrimination of *Cryptosporidium* infections. Nevertheless, detailed assessment of the genetic variation between *C. hominis* and *C. parvum* remains the most tractable approach to the provision of valuable epidemiological markers and a gene discovery vehicle for previously unnoticed genetic determinants of host specificity and virulence. To date, only one protein GP60 has shown large considerable heterogeneity between *Cryptosporidium* species and strains. This protein whose encoding gene has been used widely as a basis for strain subtyping is believed to be a surface glycoprotein subject to direct selection pressure and with a role in host–parasite interaction (Widmer and Lee 2010).

In a study by Kuo and Kissinger (2008), comparative genomic analysis of apicomplexan parasites (*Plasmodium*, *Theileria* and *Cryptosporidium*) revealed that as many as 45% of *Cryptosporidium* genes could be considered genus specific. Ordinarily, it is expected that a restriction in host range from a generalist such as *C. parvum* to a specialist such as *C. hominis* may be accompanied by selective loss of alleless; however, this study identified 334 putatively *C. hominis*-specific genes and 178 *C. parvum*-specific genes. Nevertheless, it seemed likely that this subset of genes would include the genetic determinants of phenotypic differences such as host range.

In a previous study, we also used a comparative genomic approach to identify putative species-specific genes as possible markers for host adaptation of *C. hominis* and *C. parvum* (Bouzid et al. 2010a). Our in silico analysis gave similar numbers of putatively species-specific genes to Kuo and Kissinger: 93 and 211 genes for *C. hominis* and *C. parvum*, respectively (Bouzid et al. 2010a). Unexpectedly, though, when tested experimentally, the vast majority of the predicted species-specific genes were common to *C. parvum* and *C. hominis,* in part, owing to the incomplete status of the *C. hominis* genome. Nevertheless, amongst this group of genes, experimental evidence was found for one *C. parvum*-specific gene (*Cops*-1) and one *C. hominis*-specific gene (*Chos*-1; Bouzid et al. 2010a). Interestingly, these two genes share common characteristics. Here, we describe evidence that they are founder members of a novel family of secreted telomeric contingency proteins, which may play a role in facilitating the interaction between *Cryptosporidium* and mammalian intestinal epithelium.

## Materials and methods

### Bioinformatics

*Cops-1* and *Chos-1* sequences were analysed using online software tools for protein analysis, primarily accessed via the Swiss Institute for Bioinformatics' expasy site (http://www.expasy.org/) and included kalign (http://www.ebi.ac.uk/Tools/msa/kalign/), genestream (Pearson et al. 1997), the proteincalculator v3.3 (http://www.scripps.edu/∼cdputnam/protcalc.html), interproscan (http://www.ebi.ac.uk/Tools/InterProScan/), psort ii (http://psort.ims.u-tokyo.ac.jp/), signalP3.0 (http://www.cbs.dtu.dk/services/SignalP/), radar – *R*apid *A*utomatic *D*etection and *A*lignment of *R*epeats (http://www.ebi.ac.uk/Radar/), netoglyc (http://www.cbs.dtu.dk/services/NetOGlyc/) and netnglyc (http://www.cbs.dtu.dk/services/NetNGlyc/).

### *Cryptosporidium* isolates and *Cryptosporidium* DNA

Oocysts of *C. parvum* reference strains Moredun (Moredun Institute, Scotland, UK) and Iowa II (Bunch Grass Farm, IN, USA) were obtained commercially. Purified oocysts of *C. hominis* reference strain TU502 were the kind gift of G. Widmer (Tufts Cummings School of Veterinary Medicine, MA, USA), and additional TU502 genomic DNA was supplied from ATCC-LGC standards partnership.

To test experimentally the predicted specificity of the selected genes and determine the genetic polymorphism of Cops-1 gene, a panel of purified oocysts and genomic DNAs from predominantly clinical *Cryptosporidium* isolates of *C. parvum*, *C. hominis* and *C. cuniculus* isolates was made available by the *Cryptosporidium* Reference Unit (Swansea, UK). Details of epidemiological and genotyping data from these isolates were previously described (Bouzid et al. 2010a). The clinical strains tested were *C. parvum* (Cp2, Cp3 and Cp4), anthroponotic *C. parvum* (W7265, W7266, W7267 and W7270), *C. hominis* (Ch2, Ch3 and Ch4) and *C. cuniculus* (W17330, W18455, W17525 and W17435). In addition, commercial strains Iowa, Moredun and TU502 were also tested.

### PCR screening, product sequencing and cloning and expression of the *Cops-1* gene

Primers were designed, PCR performed and PCR products sequenced using standard methods as previously described (Bouzid et al. 2010b). Full-length Cp*Cops-1* gene was amplified using the expand High Fidelity PCR system (Roche, Hertfordshire, UK) and *Cgd2_4380*_FF and FR primers (Table S1). Cloning of PCR product into cloning vector pET100/D-TOPO was achieved by topoisomerase I action allowing directional cloning. Then, the vector was transfected into a variety of competent *Escherichia coli* expression strains including DH5α, BL21 Star™ (DE3), C41 (DE3), C43 (DE3), BL21-Codon Plus-RP and Rosetta™2 (DE3) for expression purposes.

### Anti-Cops-1 monoclonal antibody

The 9E1 IgG1 mouse monoclonal antibody to *Cops-1* protein was produced by Cambridge Research Biochemicals (http://www.crbdiscovery.com). Predicted Cops-1 protein sequence (477 aa) as retrieved from NCBI (XM_626615) was submitted for peptide design, and a peptide sequence (Tyle-2) [C]-RSRPPLPTRKPYSGS-amide (position 297–311) was selected corresponding to high immunogenicity. The selected sequence was tested using Basic Localized Alignment Tools (BLASTp selected for short nearly identical oligopeptides and tBLASTn) to ensure that no sequence similarity was present for another *Cryptosporidium* species or indeed for species other than *Cryptosporidium*. This 15 amino acid sequence showed no sequence similarity. The monoclonal antibody produced was used for immunofluorescence (IFA) staining of *Cryptosporidium* oocysts. For some assays, the 9E1 was directly conjugated using Zenon® Alexa Fluor® 594 MouseIgG1 Labelling kit (Molecular Probes, Invitrogen, Paisley, UK). The specificity and staining pattern of 9E1 was tested by IFA using two *C. parvum* (Iowa, Moredun) and two *C. hominis* (Ch2, Ch4) strains.

### Antigen preparation

For Western blotting, native antigen preparation was performed from 2 × 10^6^ oocysts, concentrated by centrifugation 13 000 rpm for 10 min at 4°C. The oocysts were frozen in a dry ice/ethanol bath and immediately thawed in a water bath at 42°C. This cycle was repeated four times to ensure the breakage of the oocyst wall. Then, 2 μL of protease inhibitors was added.

### Immunofluorescence and blocking assay

For IFA, approximately 10^3^ parasites were air-dried onto single-welled microscope. Directly conjugated 9E1 antibody was incubated for 30 min at room temperature in a humidified staining chamber. After three washes in PBS, crypto-cell antibody (TCS BioSciences Ltd., Buckingham, UK) was added to the slide and incubated at 37°C for 15 min. The antibody was then carefully removed, and the slide was stained using 0.5 μg/mL 4′,6-diamidino-2-phenylindole (DAPI) for 2 min. When excystation of the oocysts was required, it was performed as previously described (Choudhry et al. 2008).

The blocking assay was conducted essentially as described by Nishikawa et al. (2000). Briefly 1 × 10^6^ *Cryptosporidium* oocysts from a purified suspension were pre-incubated with antibody (9E1) at 37°C for 90 min. As well as 9E1, a matched IgG1 isotype control (AbDSerotec; MorphoSys UK Ltd, Oxford, UK) was used. The mixture was then used to infect confluent Caco-2 cell monolayers for 3 h. The cells were washed and further cultured for 24 h before being fixed and stained with Giemsa. The effect of the pre-incubation with the antibody was assessed by parasite count for each condition from 20 randomly chosen fields.

### Patient's sera

Anonymised sera were obtained from laboratory-confirmed cryptosporidiosis cases under a study approved in 2002 (Chorley and Preston NHS Trusts Local Research Ethics Committee/Public Health Laboratory Service Ethics Committee for Investigations Involving Human Subjects) where patients gave written, informed consent for investigation of serological responses to *Cryptosporidium* infection. Patients were screened for patent *Cryptosporidium* infection by immunoblot. Positive sera showed high level of expression of the 15/17 and 27-kDa *Cryptosporidium* immunodominant proteins (R. Chalmers, Unpublished). Subsequently, *Cryptosporidium* isolates were genotyped so it was possible to differentiate between *C. parvum* and *C. hominis* serological responses as described previously (Chalmers et al. 2009). The sera used in these studies was subsequently registered, reviewed and approved (Velindre NHS Trust Research Risk Review Committee) in 2008. A negative *Cryptosporidium* patient's serum was obtained from a previous study (Elwin et al. 2007). These sera were used to test the reactivity of native *C. parvum* and *C. hominis* antigenic preparations using Western blotting, according to the standard methods essentially as previously described (Tyler et al. 2009).

## Results

### Predicted properties of the Cops-1 and Chos-1 gene products

Despite the high number of genes predicted by comparative genomics to be species specific (Kuo and Kissinger 2008; Bouzid et al. 2010a), secondary screening by PCR found that the vast majority were common to *C. parvum* and *C. hominis* (Bouzid et al. 2010a). Only two genes emerged from the secondary screen as potentially species specific; the *C. parvum*-specific gene *Cops-1* (*Cgd2_4380*) and the *C. hominis*-specific gene *Chos-1* (*Chro.50011*).

**Figure 1:**
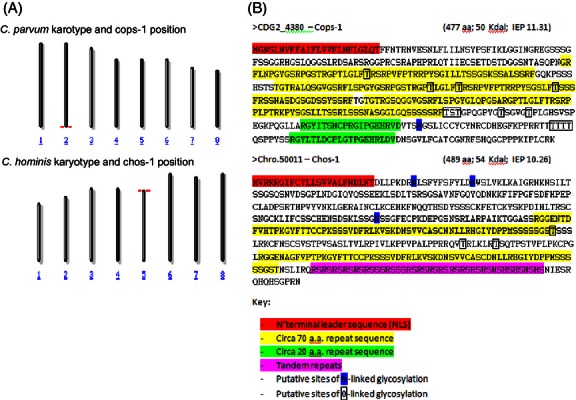
(A) *Cops-1* and *Chos-1* are positioned telomerically. The positions of each gene were mapped onto their respective genomes using the *Cryptosporidium parvum* genome and *Cryptosporidium hominis* genome views via the NCBI map viewer (http://www.ncbi.nlm.nih.gov/projects/mapview/). (B) Cops-1 and Chos-1 are predicted to encode similarly sized secreted glycoproteins. Bioinformatic analysis suggests both predicted proteins will be secreted, and both contain sites for N-linked and O-linked glycosylation. Peptides are of similar sized (*c*. 50 kDa), serine rich, contain internal repeats and are basic owing to high arginine contents.

**Figure 2:**
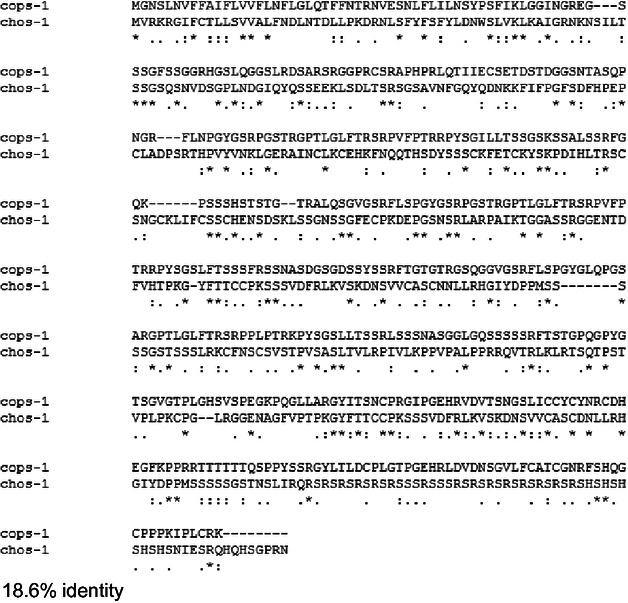
*Cops-1* and *Chos-1* show some sequence similarity. Although the internal repeats present in each protein differ, clear similarities are present across the entire length of these proteins (*E* value), suggesting that they are both members of a novel family of proteins peculiar to the *Cryptosporidium* genus.

Strikingly these two genes have shared features. Both genes were telomeric, *Cops-1* is the last gene annotated on chromosome 2 by the *C. parvum* genome project, while *Chos-1* is the first gene annotated on Chromosome 5 by the *C. hominis* genome project (Fig. 1A). Both apparently encode secreted glycoproteins with very basic core peptides of roughly 50 kDa, and both proteins contain clear but distinctive internal repeats (Fig. 1B). Remarkably, the genes show limited (18.6% sequence identity), but noticeable, sequence similarity to each other along their entire length (Fig. 2) and identified each other as the top ranked and sole significant ‘hits’ on reciprocal blasts (using tBLASTn at http://www.cryptoDB.org/) against the two genomes with *P* values of 0.001 in each case. This, taken together with the, lack of any such significant ‘hit’ against any of the other completed apicomplexan genomes identifies *Cops-1* and *Chos-1* as founder members of a novel *Cryptosporidium*-specific gene family. The initial annotation of the *Cops-1* and *Chos-1* genes provided by the *C. parvum* and *C. hominis* genome projects shows no apparent orthologs in other genomes, including *C. muris*. The limited annotation provided for *Cops-1* describes a gene encoding a serine-rich protein containing repeated motifs, with an N-terminal secretory peptide, situated proximal to the telomeric repeats of chromosome 2. The telomeric location of these genes may have hindered their detection in other *Cryptosporidium* species as telomeric regions are highly repetitive and renowned to be difficult to assemble. In *C. hominis* and *C. muris*, there are no contigs covering the *Cops-1* genomic region. In addition, the contigs in the assembled *C. hominis* and *C. muris* termini of chromosome 2 show no regions of obvious sequence similarity to the *Cops-1* gene. Similarly, the termini of chromosome 5 in *C. parvum* and *C. muris* showed no regions of sequence similarity to the *Chos-1* gene. With the exception of each other, no clear relatives for these proteins were found using sequence similarity-based searches, implying that this is a family of proteins specific to genus *Cryptosporidium*. However, interestingly, both Cops-1 and Chos-1 did show evidence for distant homology to the proteophosphoglycans of *Leishmania*.

### Cops-1 and Chos-1 species-specific amplification from a panel of strains

**Figure 3:**
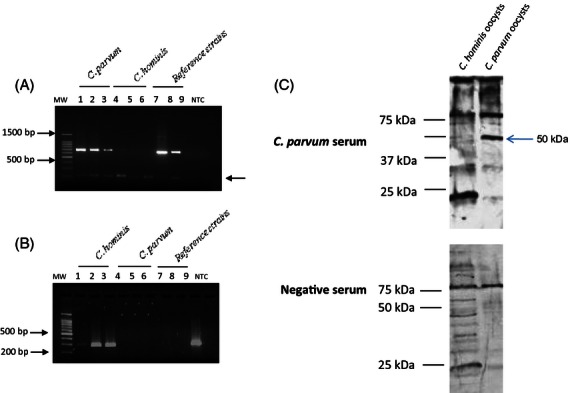
(A) Amplification of *Cops-1*: amplification of 665 bp of *Cops-1* gene from *Cryptosporidium parvum* isolates and reference strains DNA. In addition, there is a 200 bp band amplified from *C. parvum* and *Cryptosporidium hominis* DNA (arrow). 1: Cp2, 2: Cp3, 3: Cp4, 4: Ch2, 5: Ch3, 6: Ch4, 7: Iowa, 8: Moredun, 9: TU502, NTC: nontemplate control. (B) Amplification of *Chos-1*: amplification of 287 bp of Chos-1 gene from *C. hominis* clinical isolates and reference strains DNA. No amplification of *C. parvum* DNA was possible. MW, molecular weight, 1: Ch2, 2: Ch3, 3: Ch4, 4: Cp2, 5: Cp3, 6: Cp4, 7: Iowa, 8: Moredun, 9: TU502; NTC, nontemplate control. (C) A 50 kDa protein of *C. parvum* but not *C. hominis* is specifically recognized by *C. parvum-infected patient* serum. Immunoblot of antigen preparations from *C. hominis* and *C. parvum* oocysts revealed with natural *C. parvum* infection serum and controlled with negative serum. Arrow marks the *C. parvum-*specific protein.

Consistent with our bioinformatics-based predictions, when screened against clinical samples (6) and reference strains (3), the full-length gene or a 665-bp diagnostic fragment of *Cops-1* could be amplified from *C. parvum* DNA, but not from *C. hominis* DNA (Fig. 3A). Similarly, the full-length gene or a 287-bp diagnostic fragment of *Chos-1* could be amplified from *C. hominis* isolates but not from *C. parvum* DNA (Fig. 3B). *C. cuniculus,* which is known to be closely related to *C. hominis,* amplified *Chos-1* but not *Cops-1,* and *C. meleagridis* amplified *Cops-1* but not *Chos-1*; no other *Cryptosporidium* spp. which we tested (*C. andersoni*, *C. felis*, *C. ubiquitum*and *C*. *baileyi*) gave amplification of either gene (not shown). All PCR product sequences (665 bp for *C. parvum* and *C. meleagridis* and 200 bp for *C. hominis* isolates) were deposited in GenBank [HQ667112–HQ667125]. In addition, when western blots of sera from *C. parvum*-infected patients were used to probe *C. parvum* and *C. hominis* protein extracts, they revealed a *C. parvum*-specific band of the expected 50 kDa in size (Fig. 3C), supporting the supposition of *Cops-1* gene expression in *C. parvum* but not *C. hominis*.

The primers we designed for diagnostic purposes to robustly amplify the 655-bp *cops-1* gene fragment also amplified two minor bands from *C. parvum* DNA of 450 and 200 bp (Fig. 3A). This 200-bp band was also present in PCR products from *C. hominis*. Usefully, where this PCR is used diagnostically, this band can serve as an internal control for the presence of human infective *Cryptosporidium*. Interestingly, for *C. meleagridis,* PCR amplified a 665-bp product but no 450- or 200-bp bands. Thus, this assay, as it stands, confirms the presence of human infective *Cryptosporidium* and discriminates *C. hominis* from *C. parvum* and *C. meleagridis*.

In the light of the repeats contained within the *Cops-1* gene, we considered whether the 200-bp band amplified might have arisen from alternative binding sites in the *Cops-1* gene. Consistent with this, we found that the sequence obtained from both *C. hominis* and *C. parvum* showed complete (or highly similar for *C. hominis*) sequence identity to part of the *Cops-1* 665-bp PCR product, indicating alternative binding sites in the *Cops-1* gene for one of the primers and the likely presence of an orthologous gene in *C. hominis*. The gene was named Cp*Cops-1* and Ch*Cops-1* in *C. parvum* and *C. hominis*, respectively.

### An ortholog of *Cops-1* is present in *C. hominis*

As the primers used to amplify full-length *Cops-1* from *C. parvum* were unable to amplify the *C. hominis* ortholog, we tested with internal primers to determine which of the full-length primers was failing and found that the 5′ primer was able to amplify with an internal 3′ primer, but that the full-length 3′ primer could not amplify with internal 5′ primers. To amplify the full-length *C. hominis* gene, we designed a primer from the flanking region of the *C. parvum* gene. This primer, which was designed to telomeric repetitive sequence, was successful in amplifying the full-length gene from the DNA of one of the *C. hominis* clinical isolates. The full length of Ch*Cops-1* gene was determined using a primer walking approach (details of primers are shown in Table S1), and the full Ch*Cops-1* sequence is available online (GenBank HQ667126).

**Figure 4:**
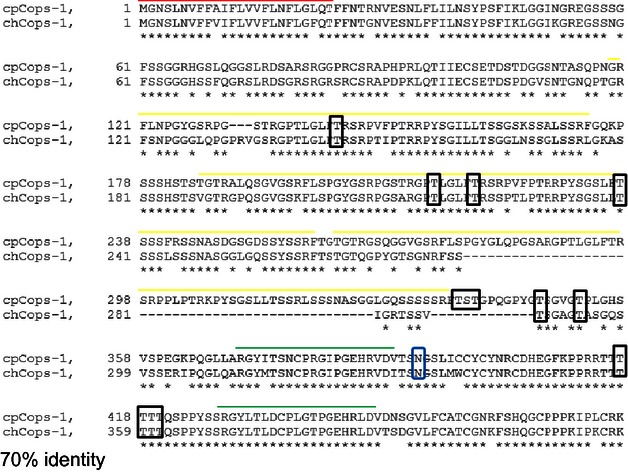
Cops-1 has an ortholog in *Cryptosporidium hominis*. In spite of being absent from the *C. hominis* genome sequence, primer walking allowed for amplification of a completed Cops-1 gene from *C. hominis* – ch*Cops*-1. This ortholog showed 70% identity with cp*Cops*-1at an amino acid level but was shorter because of the loss of approximately 70 amino acids from the third of the protein's internal repeats. Colour coding of the amino acid sequence matches that used for Cops-1 in Fig. 1B.

The full-length Cp*Cops-1* and Ch*Cops-1* are 1434 and 1263 bp, respectively. The two sequences exhibit 78.8% sequence identity. The difference in size between Cp*Cops-1* and Ch*Cops-1* corresponds to a truncation of approximately 170 bp in *C. hominis*. The alignment revealed 101 SNPs. This corresponds to an average of one SNP every 13 nucleotides. Cp*Cops-1* and Ch*Cops-1* encoded proteins are predicted to be 477 and 420 aa, respectively. The alignment of protein sequences is 70% identical (Fig. 4), with the N' terminus secretory signal peptide, and the vast majority of the putative glycosylation sites being conserved, thus maintaining the protein's general features. Based on the protein sequences alignment, 79 aminoacid substitutions were detected meaning 78.3% of SNPs are nonsynonymous.

### *Cops-1* contains sequence variation of potential taxonomic utility

**Figure 5:**
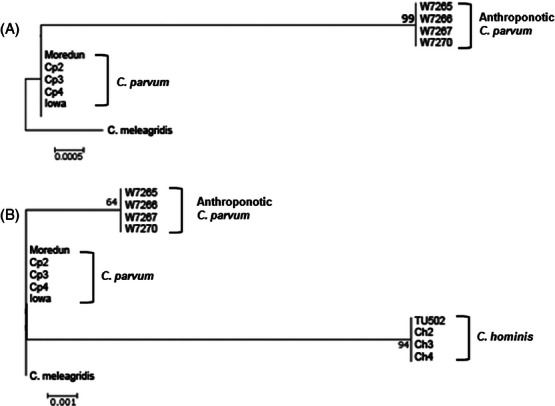
*Cops-1* sequence variation discriminates *Cryptosporidium* species and subtypes: Neighbour-joining trees of *Cops-1* sequences from *C. hominis, C. parvum* and *C. meleagridis*, based on: (A) sequences of 650-bp PCR product retrieved from *C. parvum* and *C. meleagridis*. (B) Sequences of 200-bp PCR product retrieved from *C. parvum, C. meleagridis* and *C. hominis*. The strains tested were as follows: *C. parvum* (Cp2, Cp3, Cp4), anthroponotic *C. parvum* [W7265 (W65), W7266 (W66), W7267 (W67), W7270 (W70)], *C. hominis* (Ch2, Ch3, Ch4) and *C. cuniculus* (W17330, W18455, W17525, W17435).

The observation of sequence heterogeneity between *C. parvum* and *C. hominis* at the *Cops-1* locus suggested potential taxonomic utility for the gene in subtyping of strains. Cops-1 PCR sequences from nine *C. parvum*, four *C. hominis* and one *C. meleagridis* isolates were analysed to assess the potential of this gene as a genotyping marker. The discriminatory power of Cops-1 sequence divergence was compared to genotyping using a standard *C. parvum* marker (Gp60) and a multi-locus subtyping approach (Bouzid et al. 2010a). Sequences from *C. parvum* isolates (Cp2, Cp3, Cp4, Iowa, Moredun) were identical to the published *Cgd2_4380* gene sequence. Within *C. parvum* isolates, the anthroponotic subgroup (Gp60 subtype IIc) differed by four nonsynonymous SNPs. The 200-bp *C. hominis* PCR products, despite being shorter, showed high sequence similarity to PCR products from *C. parvum* isolates. Nevertheless, the alignment revealed four *C. hominis*-specific SNPs. Of the *C. hominis* SNPs, two were synonymous and two nonsynonymous. Overall, 6/8 (75%) of the SNPs detected were nonsynonymous. Sequence analysis of *C. meleagridis* 665-bpPCR product showed only one SNP difference comparing to *C. parvum* (nonanthroponotic) sequence and that this change was nonsynonymous. The 665-bp PCR product sequences from *C. parvum* were used to build a neighbour-joining (NJ) tree, which showed good discrimination of *C. parvum* and *C. parvum* anthroponotic subtype (Fig. 5A). The sequences of the 200-bp PCR product were also used to construct a NJ tree (Fig. 5B). This tree showed that the genetic polymorphism associated with this short fragment has sufficient discriminatory power to distinguish *Cryptosporidium* genotypes and subtypes.

### Expression of recombinant Cp*C*ops-1

In expressing recombinant Cp*Cops-1* for the purposes of antibody production and serodiagnosis, we found that 11/17 (64.7%) codons had at least 20% difference in the average codon usage between *C. parvum* and *E. coli* (Fig. S1)*,* thus hindering the successful expression of the recombinant protein. Consequently, we compared expression of recombinant *Cops-1* in specialized bacterial strains, finding only two of the bacterial strains tested: BL21-Codon Plus-RP and Rosetta™2 allowed expression of the recombinant protein. Both strains have tRNAs for rare codons. The recombinant His-tagged protein observed was approximately 53 kDa, consistent with successful fusion to the N' terminal tag (Fig. S1).

### The anti-Cops-1 monoclonal antibody 9E1 does not block epithelial cell invasion by *C. parvum*

**Figure 6:**
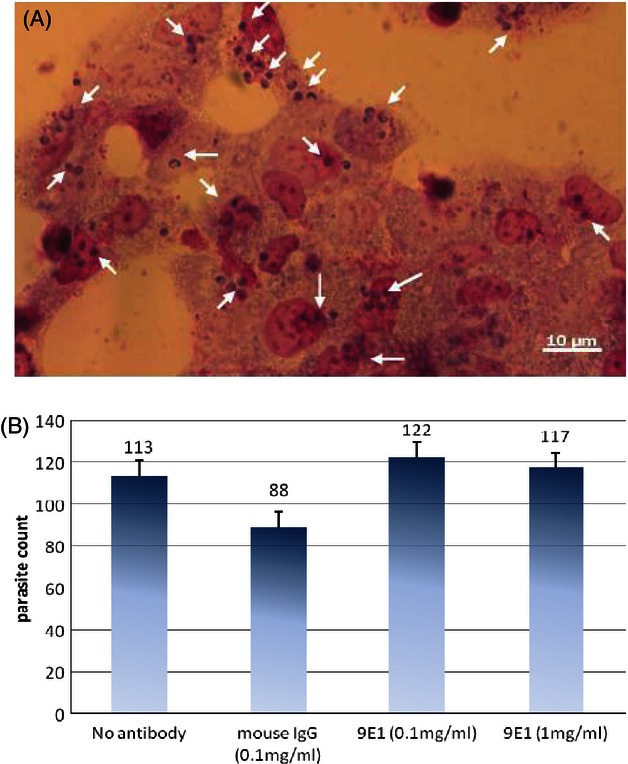
Monoclonal antibody 9E1 raised to *C*ops-1 peptide does not block intestinal epithelium cell invasion by *Cryptosporidium parvum*. Excysted *C. parvum* sporozoites were used to infect Caco-2 cell monolayers in the presence of anti-*cops*-1 monoclonal antibody. (A) Intracellular life stages of *Cryptosporidium* visualized after Giemsa staining on Caco-2 cell monolayers. (B) Mean number of parasites per field for the different coculture conditions. Pre-incubation with 9E1 monoclonal antibody did not influence the level of invasion of Caco-2 cells relative to nonspecific IgG or no-antibody controls.

The potential role of Cops-1 protein in host-cell attachment and invasion was investigated *in vitro* using a blocking assay. Purified excysted *Cryptosporidium* oocysts were cultured on Caco-2 cell monolayers, and several intracellular multiplication stages were visible from stained slides after 24 h (Fig. 6A). For the blocking assay, oocysts were pre-incubated with our anti-CpCops-1 peptide IgG1 mouse monoclonal antibody – 9E1. Control oocysts were incubated with PBS or nonspecific isotype-matched mouse IgG1 antibody. In each assay, parasite numbers were counted from 20 random fields and the mean number of parasites per field was determined (Fig. 6B). Pre-incubation of *Cryptosporidium* oocysts with 9E1 did not influence Caco-2 cells invasion. The number of parasites detected in cell monolayers following incubation of the parasites in the presence of 0.1 or 1 mg/mL 9E1 dissolved in PBS was not reduced relative to what was observed when the oocysts were incubated with PBS alone or with anisotype control (Fig. 6B).

### Cops-1 is present inside the oocyst and associated with the surface of excysting sporozoites

**Figure 7:**
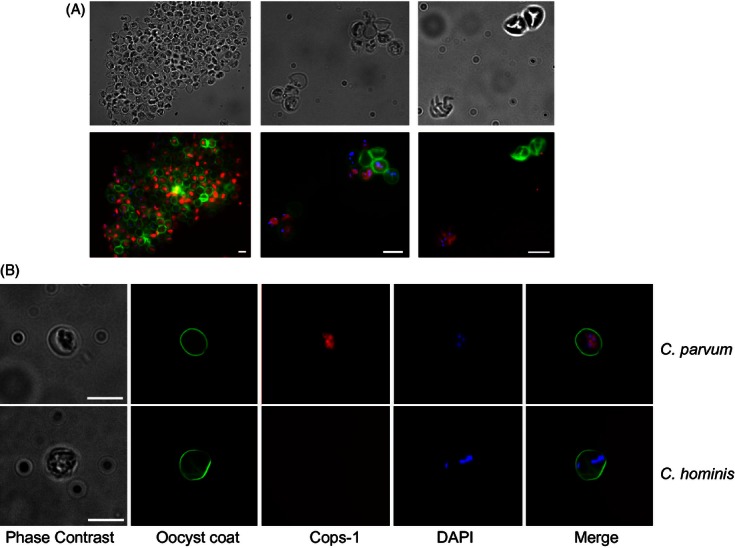
(A) Immunolocalization of *C*ops-1 protein in purified *Cryptosporidium parvum* oocysts. Left of panel shows phase-contrast microscopy of purified Moredun strain oocysts. Right of panel shows corresponding images of oocysts stained by direct IFA for *Cops*-1 with Alexa Fluor® 546 conjugated 9E1 (red) and the oocyst wall with commercial FITC-conjugated CryptoCell antibody (green) and counterstained with the DNA stain DAPI (blue). Left: on oocysts fixed and permeablized with cold methanol, 9E1 stained the contents of oocysts but not empty oocysts. Middle and right: on partial excystation of *C. parvum* oocysts and sprozoites fixed with 1% PFA but not permeablized, 9E1 stained the contents of ruptured oocysts but not intact ones and some excysted sporozoites. Scale bar = 5 μm. (B) The 9E1 monoclonal antibody to *C*ops-1 shows species specificity. 9E1 antibody shows clear staining of the oocyst contents of *C. parvum* (Iowa) oocysts (upper panel), but not of purified *C. hominis* (TU502) oocysts or oocyst contents (lower panel). Scale bar = 5 μm.

The 9E1 anti-Cops-1 antibody was also used for immunolocalization of CpCops-1 protein. For IFA, a FITC-conjugated antibody that stains the oocyst cell wall of *Cryptosporidium* species and DAPI staining of the sporozoite nuclei were used as counter stains. When oocysts were permeabilized with methanol, 9E1 antibody enabled staining of oocyst contents both intact and broken oocysts with sporozoites still inside. However, no 9E1 staining of empty oocysts was observed (Fig. 7A, left). Subsequently, we looked at staining pattern on nonpermeabilized paraformaldehyde (PFA)-fixed oocysts and found that 9E1 did not stain intact or empty oocysts. However, staining was associated with sporozoites which were emergent from the oocyst or still inside broken ones (Fig. 7A, middle) and with some free sporozoites (Fig. 7A, right). Taken together these studies suggest that Cops-1 is a secreted protein associated with the sporozoite surface and the oocyst contents.

### The 9E1 monoclonal antibody shows selective staining of purified *C. parvum* oocysts

Directly conjugated 9E1 monoclonal antibody was also used to compare staining of *C. hominis* purified oocysts with *C. parvum* purified oocysts. In contrast to *C. parvum,* the contents of *C. hominis* oocysts stained weakly or not at all (Fig. 7B). It is important to note, however, that while the antibody can discern a clear difference between *C. hominis* and *C. parvum* on highly purified permeabilized oocyst preparations, the high level of background and nonspecific staining from this monoclonal antibody on faecal samples (not shown) means that it is unlikely to be immediately useful clinically for discriminating *C. parvum* from *C. hominis* infections.

## Discussion

The Red Queen Hypothesis (Van Valen 1973) suggests a requirement for rapid co-evolution of the gene sets encoding the proteins most intimately involved in interacting with host cells: matched alleles undergoing an antagonistic co-evolution which serves to drive genetic polymorphism at key loci. We report for the first time the discovery and initial characterization of a family of telomeric proteins from *C. hominis* and *C. parvum*. The encoding genes were identified using comparative genomic analysis for divergent genes. Interestingly, the identified proteins shared common characteristics and appear to form a family of proteins with distinct properties. It is remarkable, but not altogether surprising, that this family of proteins is telomerically situated.

The telomeric location indicates that these genes are prone to higher recombination rates and are likely to encode contingency proteins. Contingency proteins are often secreted or external glycoproteins and are frequently encoded telomerically (Barry et al. 2003). Such proteins have been shown to be involved in host–pathogen interaction and parasite survival in the host with examples including the variant surface glycoprotein of *Trypanosma brucei,* which undergoes antigenic variation to evade the host immune responses and allow parasite survival (Barry et al. 2003; Yang et al. 2009), the var genes of *Plasmodium falciparum* (Kyes et al. 2001), the trans-sialidases of *Trypanosoma cruzi* (Kim et al. 2005), the major surface glycoproteins of *Pneumocystis carnii* (Benfield and Lundgren 1998) and the recently described subtelomeric variable secreted proteins of *Theileria* (Schmuckli-Maurer et al. 2009). The telomeres are prime sites for genes which are interacting with the host and evolving quickly because they are themselves dynamic and subject to novel forms of (epigenetic) regulation (Bhattacharyya and Lustig 2006; Tonkin et al. 2009; Yang et al. 2009).

In terms of size and structure, Cops-1 and *Chos-1* show similarities which suggests that they may be functionally related. As well as showing sequence similarity to each other, they show some similarity to the phosphoproteoglycans of *Leishmania* which are secreted glycoproteins that have been shown to interact with the host immune system (Ilg et al. 1995; Piani et al. 1999).

Our comparative genomic approach aimed to identify coding loci with species-specific characteristics with a view to their exploitation for diagnosis and discrimination of human infective *Cryptosporidium* species. Given the considerable similarity of the vast majority of coding sequences between *C. parvum* and *C. hominis*, the genetic heterogeneity displayed by these proteins is itself an indicator that these genes are likely to be under direct selective pressure for adaptation through interaction of the proteins they encode with the host cells. The adaptation of these proteins most likely reflects the characteristics of host–pathogen interaction as a preferred niche and thus may contribute directly to the parasite's ability to colonize and infect particular hosts.

In the case of *Cops-1*, *in silico* prediction of species specificity was rejected experimentally when our analysis suggested the presence of an abridged ortholog is present in *C. hominis* (Ch*Cops-1*). Sequencing of a small PCR product from *C. hominis* showed high sequence similarity to *Cops-1* providing evidence for a previously undiscovered ortholog of this gene in *C. hominis* (Ch*Cops-1* has eluded *C. hominis* genome project). The exact genomic location of this gene is unknown, although it is likely to be telomeric as the primer which was used to amplify the full-length gene was designed to hybridize telomeric repeats. The two genes were named Cp*Cops-1* and Ch*Cops-1*, for *C. parvum* and *C. hominis*, respectively. Comparative Southern blot analysis is likely to prove the definitive approach to assessing the presence, absence and positioning of orthologs of these genes in different *Cryptosporidium* species and strains. This approach is, however, hindered, particularly for *C. hominis,* by the difficulties in producing large amounts of genomic DNA from organisms which do not proliferate readily or substantially using current culture protocols.

Our PCR for *Cops-1* is useful as a diagnostic test, specifically amplifying a 655-bp product from *C. parvum* isolates and a 200-bp product from both *C. hominis* and *C. parvum* DNA. Sequencing of the 200-bp fragment enabled limited subtyping; indeed, a phylogenetic tree drawn from the sequence variation observed in this short fragment had a good discriminatory power and allowed discrimination of *Cryptosporidium* genotypes and subtypes, which is consistent with the previous multi-locus analysis, therefore suggesting comparable polymorphism levels between this fragment and other genetic loci (Bouzid et al. 2010a). We successfully expressed the recombinant CpCops-1 protein raising the prospect for its use for serodiagnosis.

Perhaps unsurprisingly, our anti-CpCops-1 monoclonal antibody (9E1) seems to be a nonblocking and nonneutralizing antibody, and this may simply imply that Tyle-2 epitope is not located in a region interacting with the intestinal cell receptors. When used for immuno-localization studies in faecal samples, the high background staining and relatively low intensity of the *C. parvum*-specific staining with 9E1 also meant that the antibody was unlikely to serve routinely as a useful diagnostic test to discriminate *C. parvum* from *C. hominis*. On purified *C. parvum* oocysts and sporozoites, though, the monoclonal antibody clearly recognized the contents of the oocyst when permeabilized and was able to stain free sporozoites without permeablization, demonstrating the association of CpCops-1 with the sporozoite surface. Conversely, on *C. hominis* oocysts and sporozoites, 9E1 showed little or no staining.

We have been unable to find evidence for expression of ChCops-1 by *C. hominis* either experimentally or in published databases. At point of publication it has not been reported in any of the several transcriptomic and proteomic data sets available for *C. hominis*. The inability of our antibody to detect the protein in *C. hominis* may reflect lack of expression but could also be due to differences in antigenicity between the orthologs. Our immunoblot of native *C. parvum* and *C. hominis* antigenic preparations probed with *C. parvum*-specific patient sera revealed an apparently *C. parvum*-specific antigen of the expected size for cpCops-1 but not for ChCops-1. If this antigen-specific band does accord with CpCops-1, it suggests that the *C. hominis* ortholog is either sufficiently dissimilar antigenically likewise not to be recognized by the patient sera or alternatively simply not expressed. Irrespective of the relative expression of these family members, the novel characteristics, localization and structural characteristics of this *Cryptosporidium-*specific family of proteins make Cops-1 and Chos-1 not only diagnostically and taxonomically useful, but plausible candidates as mediators of host specificity and virulence.

## Data archiving statement

Genomic data for this study are available on Genbank, and accession numbers are provided in the manuscript.

## Supporting Information

**Figure S1.** Cops-1 shows a strong bias in codon preference necessitating specialised strains of *E. coli* for expression of recombinant protein.

**Table S1.** Details of the primers used to amplify Cops-1 gene.
