# Supplementary material for: A new heterogeneous family of telomerically encoded Cryptosporidium proteins
Source: Evol Appl. 2012 Jun 14;6(2):207–17. doi: 10.1111/j.1752-4571.2012.00277.x (PMC3586618; doi:10.1111/j.1752-4571.2012.00277.x)
Supplement: Supplementary file 1 [file eva0006-0207-SD1.ppt]

## Slide 1
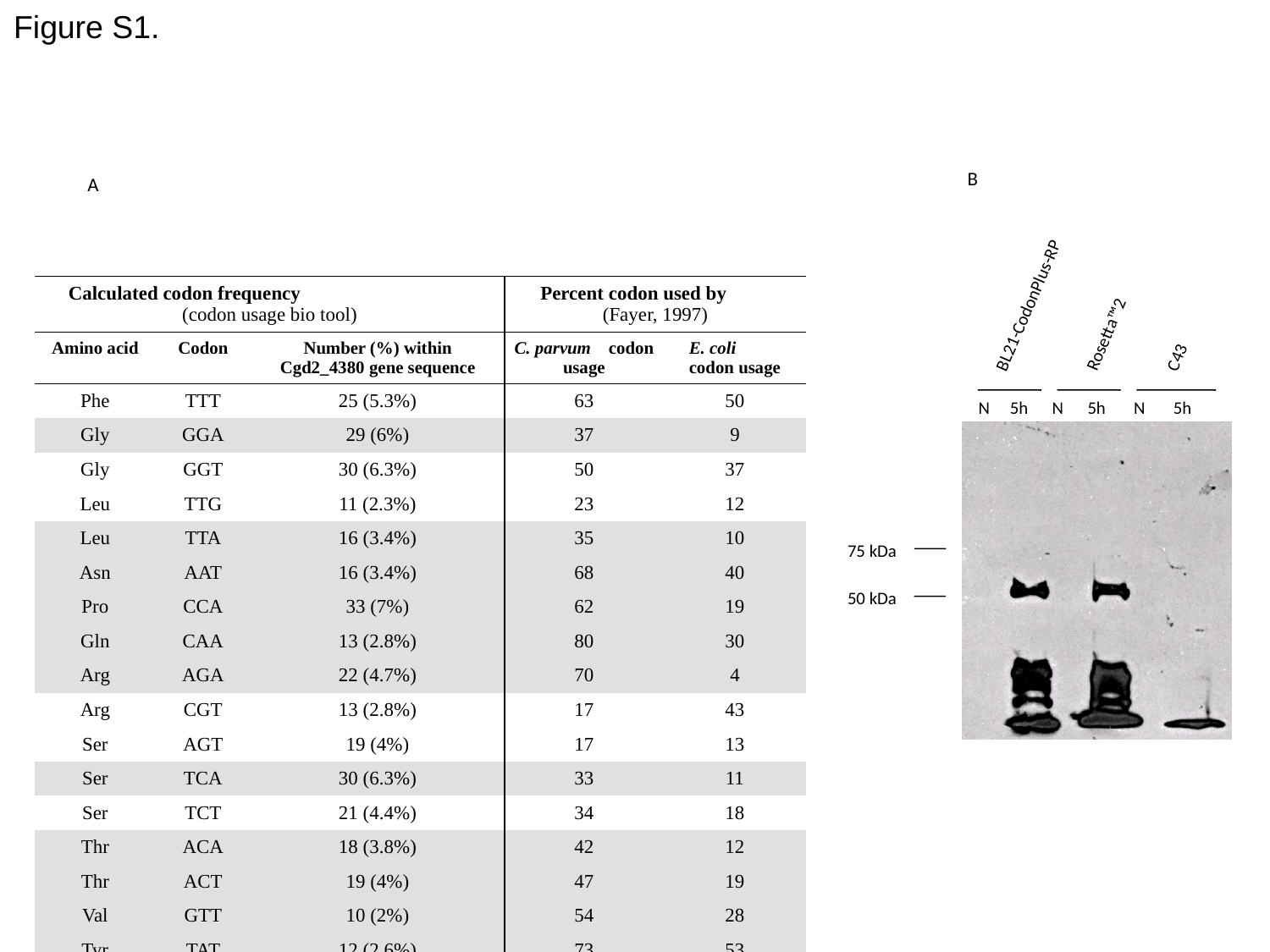

Figure S1.
 N 5h N 5h N 5h
75 kDa
50 kDa
B
A
| Calculated codon frequency (codon usage bio tool) | | | Percent codon used by (Fayer, 1997) | |
| --- | --- | --- | --- | --- |
| Amino acid | Codon | Number (%) within Cgd2\_4380 gene sequence | C. parvum codon usage | E. coli codon usage |
| Phe | TTT | 25 (5.3%) | 63 | 50 |
| Gly | GGA | 29 (6%) | 37 | 9 |
| Gly | GGT | 30 (6.3%) | 50 | 37 |
| Leu | TTG | 11 (2.3%) | 23 | 12 |
| Leu | TTA | 16 (3.4%) | 35 | 10 |
| Asn | AAT | 16 (3.4%) | 68 | 40 |
| Pro | CCA | 33 (7%) | 62 | 19 |
| Gln | CAA | 13 (2.8%) | 80 | 30 |
| Arg | AGA | 22 (4.7%) | 70 | 4 |
| Arg | CGT | 13 (2.8%) | 17 | 43 |
| Ser | AGT | 19 (4%) | 17 | 13 |
| Ser | TCA | 30 (6.3%) | 33 | 11 |
| Ser | TCT | 21 (4.4%) | 34 | 18 |
| Thr | ACA | 18 (3.8%) | 42 | 12 |
| Thr | ACT | 19 (4%) | 47 | 19 |
| Val | GTT | 10 (2%) | 54 | 28 |
| Tyr | TAT | 12 (2.6%) | 73 | 53 |
