# Supplementary material for: A new heterogeneous family of telomerically encoded Cryptosporidium proteins
Source: Evol Appl. 2012 Jun 14;6(2):207–17. doi: 10.1111/j.1752-4571.2012.00277.x (PMC3586618; doi:10.1111/j.1752-4571.2012.00277.x)
Supplement: Supplementary file 2 [file eva0006-0207-SD2.docx]

**Supporting Information**

**Figure S1:**

***Cops*-1 shows a strong bias in codon preference necessitating specialised strains of *E. coli* for expression of recombinant protein. A:** Codons present at a frequency ≥ 2% in the *Cops*-1 gene as determined by the codon usage software. The percentage was calculated based on the total number of 478 trinucleotides (including stop codon). The average of codon usage in percentage was adopted from Fayer (1997). The highlighted codons showed over 20% difference between *C. parvum* and *E. coli* codon usage. **B:** Comparison of the level of expression of the his-tagged recombinant *Cops*-1 protein between the three specialized strains (BL21-CodonPlus-RP, Rosetta™2 and C43 strains) 5h post induction. BL21-CodonPlus-RP and Rosetta™2 strains exhibited the same level of expression, while C43 strains failed to express the recombinant protein.

Table S1: Details of the primers used to amplify *Cops-1* gene

| **Primer name** | **Sequence** | **Gene position** | **Tm (^◦^C)** |
| --- | --- | --- | --- |
| Cgd2_4380 F | AAGGGGTGGACCTAGATGCT | 243-262 | 62.45 |
| Cgd2_4380 R | GAAGAGGTGGGCGTGATCTA | 888-907 | 62.45 |
| Cgd2_4380FF | ATGGGTAATAGTTTAAATGTTTTT | 1-24 | 52.61 |
| Cgd2_4380FR | TTATTTTCGGCATAACGG | 1417-1434 | 53.07 |
| Cgd2_4380_2F | GGGGTGGACCTAGATGCTC | 245-263 | 64.48 |
| Cgd2_4380_2R | GAGGTGGGCGTGATCTAGTAAA | 883-904 | 62.67 |
| Cgd2_4380_flanking_5’end | AAAAGCGCAAGTAATCTGGA | Upstream sequence | 56.3 |
| Cgd2_4380_flanking_3R | CCTAAACCTAACCCCCTAAACCT | Downstream sequence | 62.77 |
